# Carbohydrate Synthesis is Entering the Data‐Driven Digital Era

**DOI:** 10.1002/chem.202500289

**Published:** 2025-04-18

**Authors:** Eric T. Sletten, Jakob B. Wolf, José Danglad‐Flores, Peter H. Seeberger

**Affiliations:** ^1^ Max Planck Institute of Colloids and Interfaces Potsdam Science Park Am Mühlenberg 1 14476 Potsdam Germany; ^2^ Institut für Chemie, Biochemie und Pharmazie Freie Universität Berlin Takusstraße 3 14195 Berlin Germany

**Keywords:** automation, digital chemistry, glycosylation

## Abstract

Glycans are vital in biological processes, but their nontemplated, heterogeneous structures complicate structure‐function analyses. Glycosylation, the key reaction in synthetic glycochemistry, remains not entirely predictable due to its complex mechanism and the need for protecting groups that impact reaction outcomes. This concept highlights recent advancements in glycochemistry and emphasizes the integration of digital tools, including automation, computational modelling, and data management, to improve carbohydrate synthesis and support further progress in the field.

## Graphical abstract

The outcome of glycosylations, essential for glycochemistry, is difficult to predict due to its intricate mechanism and the influence of protective groups. Recent advances illustrate that integrating digital tools like automation, computational modelling, and data management drives progress in the carbohydrate synthesis field.

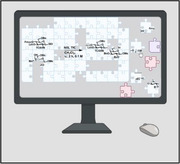

## 1 Introduction

Glycans are essential for a wide range of biological processes.^[^ ^1^ ^]^ From serving as an energy source to playing roles in pathogen interactions, biomaterial scaffold formation, and cell‐cell signaling, carbohydrates are integral to nearly all aspects of biology.^[^ ^2^ ^]^ Understanding the structure‐function relationship of glycans is complicated by their inherent nontemplated nature.

**Figure 1:**
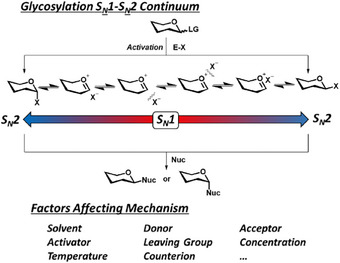
Pathway of glycosylation mechanism along a S_N_1 to S_N_2 continuum and contributing factors.

Advances in synthesis and modification of homogenous glycan motifs must keep pace with recent breakthroughs in glycobiology in order to provide tools for detailed investigations.^[^ ^3^, ^4^, ^5^, ^6^, ^7^, ^8^, ^9^ ^]^ The key chemical reaction of the glycosciences, the glycosylation, suffers from a long‐standing reputation for unpredictability and irreproducibility.^[^ ^10^ ^]^ The reaction mechanism of glycosylations follow paths along a S_N_1 to S_N_2 continuum depending on the influence of various continuous and discrete conditional factors that influence the stereochemical outcome (Figure 1).^[^ ^11^ ^]^ The presence of multiple hydroxyl groups in carbohydrates provides different positions of elongation, necessitating the use of protecting groups to achieve regioselectivity. The protecting groups are not merely passive bystanders in the reaction but have significant impact on the stereoelectronic properties of the molecule. These intrinsic problems of chemical glycosylation can be circumvented by enzymatically or chemoenzymatically constructing complex glycans without the need of protecting groups.^[^ ^12^, ^13^, ^14^, ^15^, ^16^, ^17^, ^18^, ^19^, ^20^ ^]^ However, these enzymatic methods are limited in substrate scope.

Here, we describe the current state of glycochemistry and highlight the application of new digital tools to the glycosciences. We provide suggestions as for a better integration of automation, computational tools, and data management to further enhance carbohydrate synthesis.

## 2 Current State of Glycochemistry

Before applying digital tools to glycochemistry, it is essential to identify the existing challenges in glycosynthesis.^[^ ^21^ ^]^ Assembling the glycosynthesis puzzle with the help of digital tools requires systematic assignment of the pieces and identifying the missing parts of the grand picture. Recent developments in chemical synthesis methodologies for glycan construction have focused on the synthesis of *C*‐glycosides, encompassing both alkyl and aryl derivatives.^[^ ^22^, ^23^, ^24^, ^25^, ^26^, ^27^, ^28^, ^29^ ^]^ Despite numerous syntheses of complex, bespoke glycans,^[^ ^30^, ^31^, ^32^, ^33^ ^]^ significant challenges in chemical *O*‐glycoside methodology remain unresolved.^[^ ^21^ ^]^ Without a centralized, comprehensive glycosylation database, information on chemical glycosynthesis is mostly scattered throughout the scientific literature, shared anecdotally, or exists merely as tacit knowledge of versed practitioners. Novices to the carbohydrate field face challenges that result in reproducibility issues.^[^ ^34^ ^]^ Fundamental mechanistic insights, essential for predicting novel glycosylation reactions, are obscured by incomplete data.^[^ ^11^, ^35^ ^]^ Without a standard approach, each individual glycosylation is treated as a unique coupling, with the choice of protecting groups, leaving groups, and reaction conditions such as reaction temperature, time, solvent choice, and concentration based on experience. The lack of uniformity and completeness in data reporting is a significant barrier to data‐driven models. Arbitrary parameters, such as ill‐defined temperature ramps or vague reaction times (e.g., “overnight”), are often used, making it difficult to identify precise conditions.^[^ ^34^ ^]^

Crich illustrated the extensive variety of glycosylation conditions in 2011, referencing Hindsgaul's 1995 tabulation of over 700 glycosylation reactions, promoters, solvents, and temperatures involved, highlighting the significant empiricism and the complex terminology of the field.^[^ ^36^, ^37^ ^]^ An updated tabulation of 2010 data was considered to show similar diversity, a situation that likely has not changed until today. However, new automated platforms and digital tools to handle large chemical datasets can leverage the empirical nature of glycosylation research. While these algorithms and tools have existed for decades,^[^ ^38^, ^39^, ^40^ ^]^ accessibility and ease of use have improved recently. Widespread application has demonstrated the value of addressing current synthetic challenges using algorithms that blur the boundaries between chemical synthesis and data science.^[^ ^41^, ^42^, ^43^, ^44^, ^45^ ^]^

## 3 Prerequisites for Digital Glycoscience

**Figure 2:**
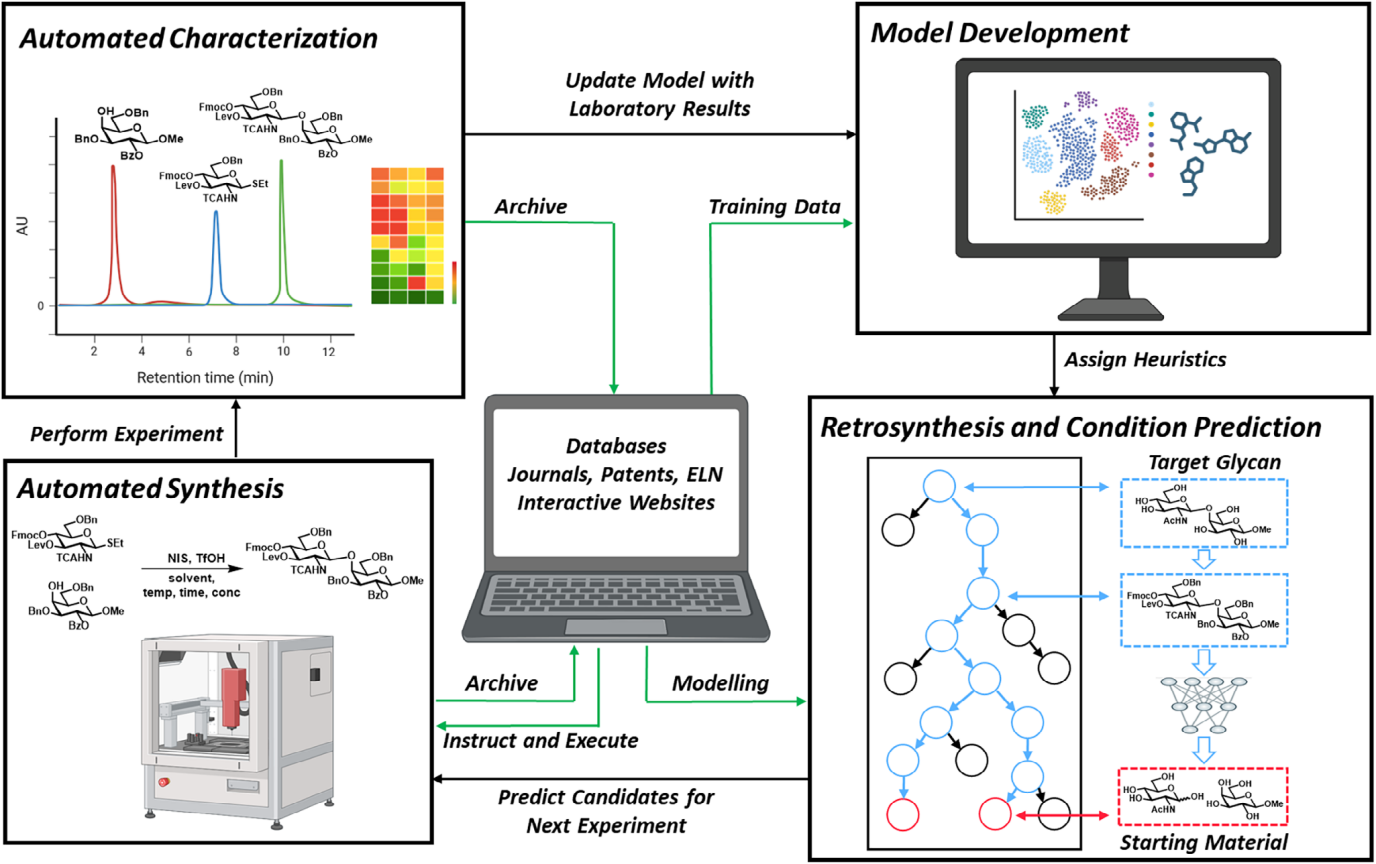
Potential workflow for the advancement of glycosylation chemistry using digital chemistry tools.

Digital chemistry applies computational tools, algorithms, and digital technologies to solve chemical challenges by simulating, analyzing, accelerating, and predicting chemical processes and molecular behavior. It enables deeper insights into molecular interactions, reaction mechanisms, and material properties through data‐driven modeling and automation, enhancing both research efficiency and discovery potential across fields such as drug development, materials science, and sustainable chemistry (Figure 2).^[^ ^44^, ^46^ ^]^ Key focus areas include quantum mechanical calculations, molecular modeling, cheminformatics, virtual screening, machine learning, and artificial intelligence applications, as well as specialized tools and software like electronic lab notebooks, searchable online repositories, retrosynthetic planning applications, and robotics.^[^ ^44^, ^46^ ^]^

While some of these methods, such as simulations or automated screenings, create large amounts of data, others, such as machine learning for retrosynthetic planning, require large amounts of data. For data to be usable or understandable for different people or applications, it has to contain metadata, to record and specify what was measured under which conditions. This data needs to be structured in such a way that it is easily reusable. FAIR data is the core of digitalization and scientific data management as data stewardship should be based on four foundational values: Findability, Accessibility, Interoperability, and Reusability.^[^ ^47^ ^]^ Reporting FAIR data facilitates and simplifies the processes of discovery, evaluation, and reuse in subsequent studies. Widespread implementation of the FAIR principles will help bridge the information gap by data standardization, reproducibility, and evaluation.^[^ ^48^ ^]^ In the past, limited page counts in print publications prevented most scientific data from being published, but even today, despite the capacity of digitally capturing extensive amounts of data, much data remain unpublished or difficult to reuse.^[^ ^49^ ^]^ To advance data‐intensive glycoscience research and address reproducibility issues, we must change how experimental data are collected and reported, ensuring it is both structured and open, and complemented by tools like semantic web technologies to enhance understanding and usability.^[^ ^49^ ^]^ Implementation of Laboratory Information Management Systems (LIMS) and Electronic Lab Notebooks (ELN), represented by open‐source solutions such as OpenBIS or Chemotion, can help this objective.^[^ ^49^, ^50^, ^51^ ^]^ Automation and real‐time reaction monitoring enable data‐rich experimentation, crucial for navigating the complexities of chemical synthesis.^[^ ^45^, ^52^, ^53^, ^54^ ^]^ Flexible, automated, and high‐throughput experimentation (HTE) platforms help minimize human error and provide an unbiased representation of both positive and negative results.^[^ ^48^, ^55^, ^56^, ^57^ ^]^ These platforms are expensive and experimentation can be performed manually in well‐plates as well. However all metadata, including the reaction setup, must be reported to be useful.^[^ ^10^, ^48^, ^58^ ^]^ Publishing the data in searchable and machine‐actionable online repositories such as the Open Reaction Database (ORD) or Chemotion, ensures that information is findable and interpretable by chemists and their computational agents.^[^ ^49^, ^50^, ^59^, ^60^ ^]^ Standardized data structuring paves the way for data‐driven techniques, like machine learning (ML) and artificial intelligence (AI), to become indispensable tools for scientific advancement.^[^ ^44^, ^61^ ^]^

**Figure 3:**
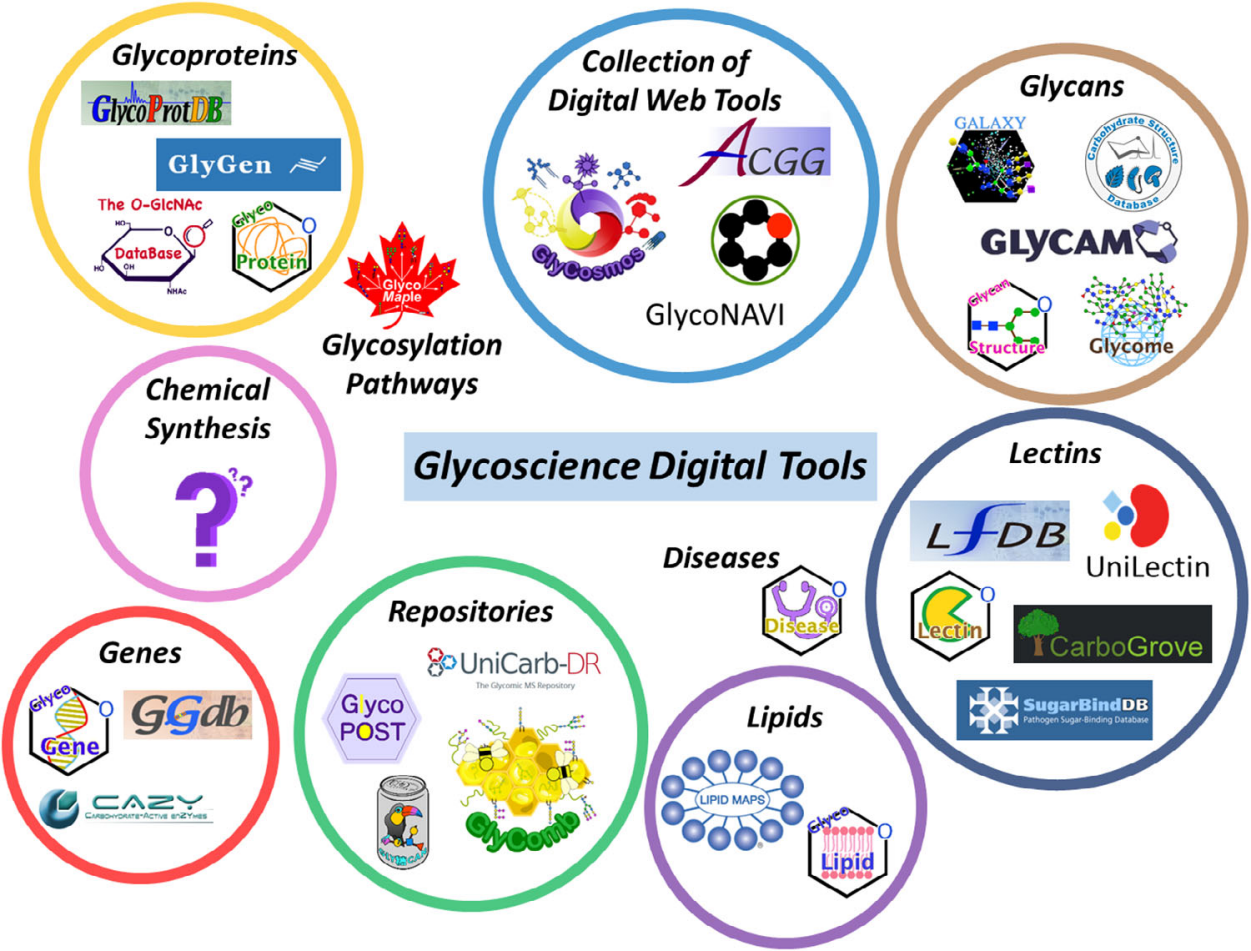
Different categories of glycoscience digital tools utilized to accelerate discovery and understanding.

Mid‐sized context‐sensitive databases and ontologies were developed early for the glycosciences (Figure 3). CarbBank, a database project for glycan structures was established in 1987 and contained over 23000 glycan sequences.^[^ ^62^ ^]^ Multiple other database projects followed, including the Kyoto Encyclopedia of Genes and Genomes (KEGG) Glycan,^[^ ^63^ ^]^ the database of the US Consortium for Functional Glycomics (CFG),^[^ ^64^ ^]^ GLYCOSCIENCES.de,^[^ ^65^ ^]^ GlycoSuiteDB,^[^ ^66^ ^]^ UniCarbKB,^[^ ^67^ ^]^ Carbohydrate Structure Database (CSDB),^[^ ^68^ ^]^ GlycomeDB,^[^ ^69^ ^]^ and EUROCarbDB.^[^ ^70^ ^]^ By implementing Semantic Web technologies, GlyTouCan linked glycan structures across multiple glycomic databases by a unique accession number.^[^ ^71^ ^]^ GlycoRDF, a Resource Description Framework (RDF) for a standard representation of storing glycomics data (glycan structures, publication information, biological source information, and experimental data) simplified this process.^[^ ^72^ ^]^ Glycobiology repositories and databases are compiled on GlyCosmos (https://glycosmos.org/) for structure‐property relationship queries.^[^ ^73^ ^]^ Leveraging the technologies summarized in Figure 3, ensured that glycan data is accessible, interoperable, and useful for researchers and machines.

## 4 Toward Digitizing Chemical Glycan Synthesis

Databases describing the chemical synthesis of glycans are missing (Figure 3). The key is standardization of the discrete input data (reagents, donor, acceptor, activator, solvent, reaction equipment, and setup), and continuous reaction parameters (time, temperature, concentration), mapped to the raw (chromatograms and spectra containing sufficient metadata) and interpreted output data (yield, selectivity, conversion). The databases should follow the guidelines put forth by Andreana and Crich.^[^ ^34^ ^]^ A glycosylation‐specific ontology would facilitate the development of a semantic web‐based repository for the curation of chemical species, their properties, and reactions. Thereby, it will be possible to examine the breadth and coverage of the glycosylation space^[^ ^74^ ^]^ and correlate yield, stereoselectivity, and conversion with a specific structural (steric or electronic) or underlying property using quantitative structure–activity relationship models (QSAR models).^[^ ^75^ ^]^ Stereoselectivity, solvent effects, armed/disarmed theory, and long‐range participation could be quantified.^[^ ^11^, ^35^, ^75^, ^76^, ^77^, ^78^ ^]^ Since the majority of practitioners invest the majority of their time in the construction of monosaccharide building blocks, detailed synthetic information should encompass protecting group manipulations on glycosyl donors and acceptors. The information will provide valuable insights into retrosynthetic routes to afford monosaccharides and protecting group orthogonality.^[^ ^79^ ^]^ Within the realm of automated synthesis, a consistent format for reporting operational protocols is still missing, in particular for carbohydrate synthesis;^[^ ^13^, ^32^, ^80^, ^81^, ^82^, ^83^, ^84^, ^85^, ^86^ ^]^ beyond a detailed setup description, effective sharing of the steps and parameters is pivotal for ensuring reproducibility and standardization of the procedures through the field.

Automated experimentation to rapidly and uniformly explore chemical space has been the basis to standardize data formats.^[^ ^74^, ^87^ ^]^ HTE datasets are available for few reaction types, most commonly Buchwald^–^Hartwig aminations and Suzuki couplings, however more diverse datasets are emerging.^[^ ^42^, ^43^, ^88^ ^]^ Extensive reaction datasets as the Chemical Abstracts Service (CAS) Content Collection, Pistachio, USPTO, and Reaxys contain numerous reactions but are proprietary, have varied protocols, and/or underrepresent negative results and can be skewed toward popular reactions and/or methodologies.^[^ ^48^, ^55^, ^56^, ^57^, ^89^, ^90^, ^91^, ^92^ ^]^ Reaction‐specific, rigorous, well‐defined repositories, such as those developed by AbbVie for C─C cross‐couplings, help mitigate the impact of reaction condition variations.^[^ ^91^, ^92^ ^]^ Similarly, in computer‐aided retrosynthesis, large reaction datasets are significantly enhanced by integrating synthesis expert knowledge.^[^ ^93^ ^]^ These initiatives underscore the importance of developing comprehensive and standardized datasets and digital tools to advance the field effectively.

The application of digital tools for glycochemistry such as data visualization or AI/ML for reaction condition prediction and retrosynthetic models is challenging. Building glycosylation reaction databases bottom‐up requires numerous experiments. A community effort is needed as reagent procurement is time‐consuming. Structural configuration, protecting groups, and chain length of carbohydrate building blocks profoundly impact sterics, electronics, and thereby the reactivity.^[^ ^94^ ^]^ Although there are only ten types of monosaccharides in mammalian systems,^[^ ^12^ ^]^ the inclusion of all the permutations of protecting groups at each position results in near‐infinite combinations. The incorporation of bacterial motifs would result in a significant increase in the number of monosaccharide types and required protected building blocks. However, the sets of glycosyl pairs trialed can be minimized to effectively and efficiently traverse the glycosylation chemical space by balancing exploration and exploitation.^[^ ^74^ ^]^ The representation of protected glycan structures in both a human‐readable, typically pictorial chair representation, and a machine‐readable form (e.g., SMILES) simultaneously needs development. Currently, no grammar‐based language exists that can be intuitively and rapidly written and understood by both human and machine, such as the IUPAC condensed form used by GlyLES which is limited to unprotected structures.^[^ ^95^ ^]^ The experimental means of data acquisition and the platform used are important. Traditional reaction conditions for glycosylations are not directly amenable to commercial HTE systems, due to the necessity for anhydrous conditions, sub‐zero temperatures, and the use of volatile solvents such as methylene chloride. Rapid reaction rates further complicate the acquisition of kinetic data, all those points requiring construction of bespoke in‐house systems.^[^ ^78^ ^]^

## 5 Digitalizing Glycochemistry: Current State of the Art

**Figure 4:**
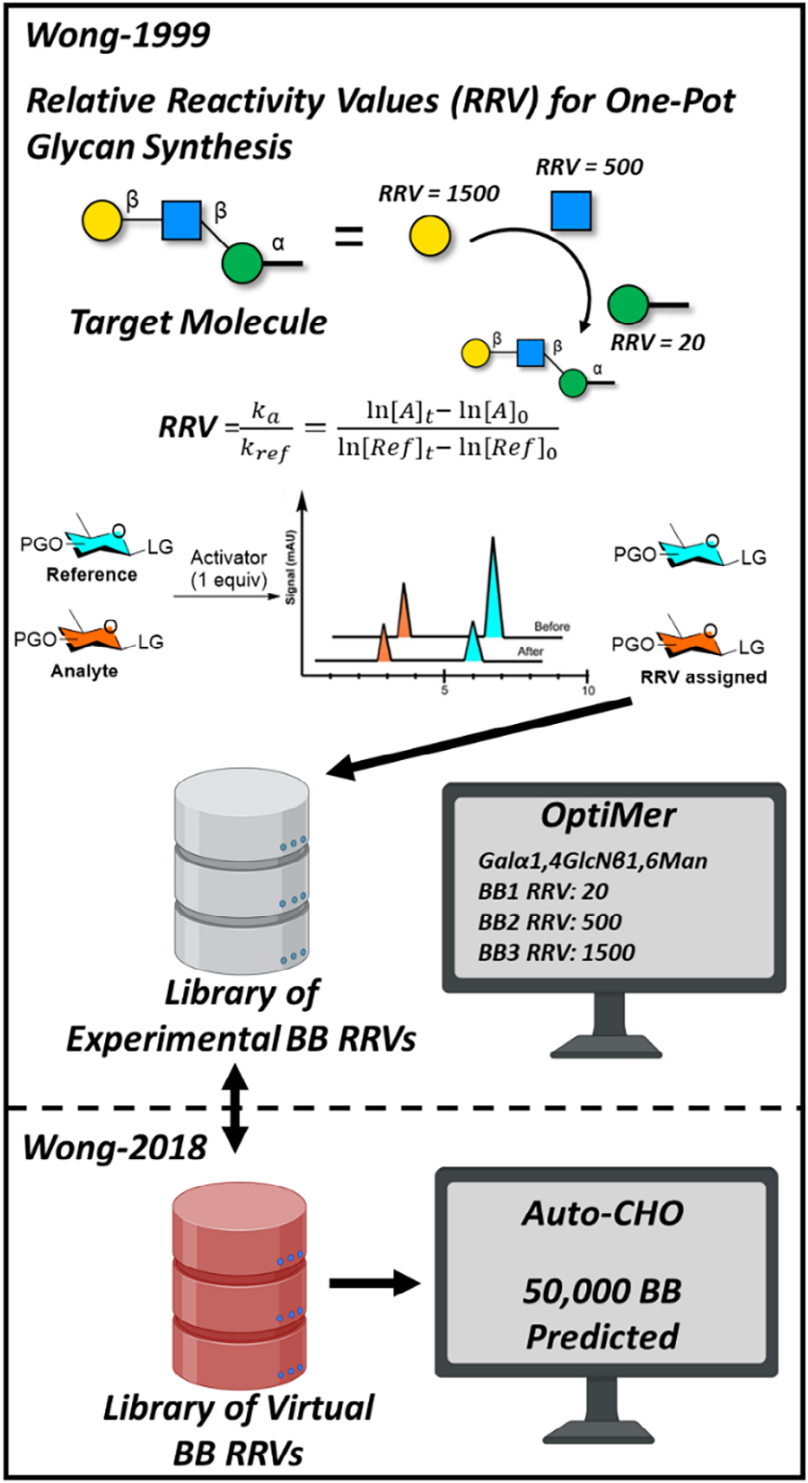
Database software tools for one‐pot glycan construction retrosynthesis based on RRVs.

Sophisticated digital tools and technologies (Figure 4) have been developed to address some of the challenges described above and discussed by others in the field.^[^ ^82^ ^]^ In 1999, the OptiMer software for programmable one‐pot oligosaccharide synthesis was developed by Wong.^[^ ^96^ ^]^ The OptiMer software filters all the donors in a database for sugar types, glycosylation sites, and stereochemical properties to predict the best donor sets for a target sequence by looking at their respective relative reactivity values (RRV). Using deterministic and Monte Carlo methods, suitable donor combinations and synthetic routes that maximize RRV differences were identified. In contrast, experts consider practical factors like availability and cost when selecting the final route. The limitations of the OptiMer software were addressed with the introduction of Auto‐CHO.^[^ ^97^ ^]^ The new tool employs ML to predict the RRVs of 50000 virtual building blocks (BBL) via generation of 1D and 2D molecular descriptors from the original dataset.^[^ ^94^, ^98^ ^]^

**Figure 5:**
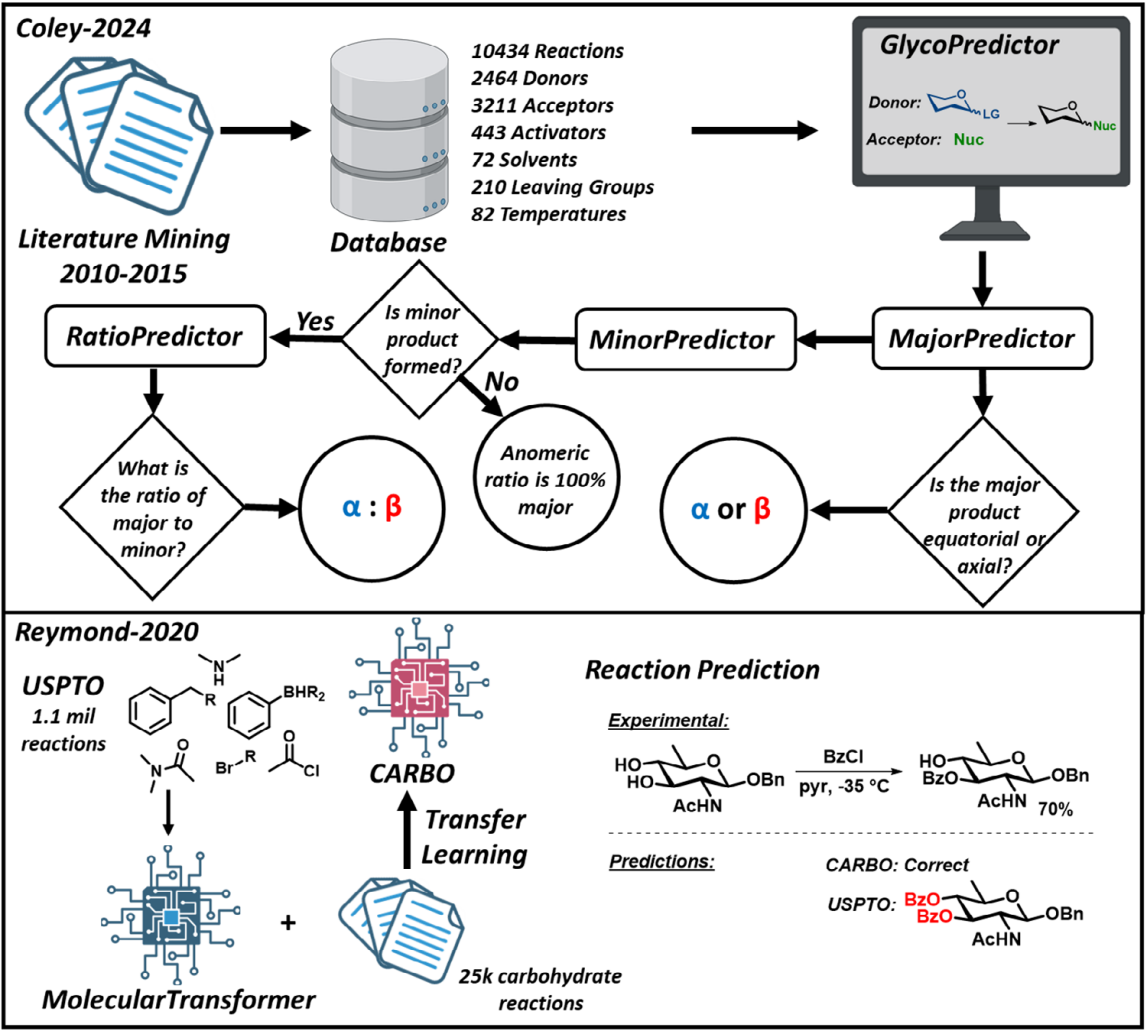
Carbohydrate reaction prediction mining literature data.

In an attempt to train models on literature datasets, Coley extracted over 10000 glycosylation reactions from the CAS Content Collection and the Hindsgaul dataset with the objective of creating machine‐learnt models (Figure 5).^[^ ^36^, ^99^ ^]^ These models predict the predominantly formed anomer in a glycosylation reaction, determine whether the other anomer also forms and if so, estimate the anomeric ratio. The resulting models have been incorporated into a publicly accessible tool, named GlycoPredictor.^[^ ^99^ ^]^ Similarly, Reymond applied transfer learning to the *Molecular Transformer*, developed earlier by Schwaller et. al. for general reaction prediction based on the USPTO database^[^ ^100^ ^]^ by refining it with 25,000 manually extracted carbohydrate reactions from the Reaxys database.^[^ ^101^ ^]^ The refined CARBO model was able to successfully predict the synthesis of a trisaccharide with five challenging regioselective protections and four difficult regio‐ and stereoselective glycosylations, with 68% of steps correctly predicted, as opposed to only 39% by *Molecular Transformer*. The authors attributed the majority of erroneous predictions of CARBO to deficiencies in the training data, including the absence of stoichiometry. The development of more accurate glycosylation models hinges on the creation of an open database of reaction data, accompanied by comprehensive machine‐readable metadata associated with each transformation. Similarly, the Coley model, yielding an *R* ^2^ of 0.58 for anomeric prediction would benefit from curated datasets. Accurate glycosylation models depend on accurate data, calling for the creation of a machine‐readable, open database of glycosylation data, annotated by comprehensive metadata for each transformation.

**Figure 6:**
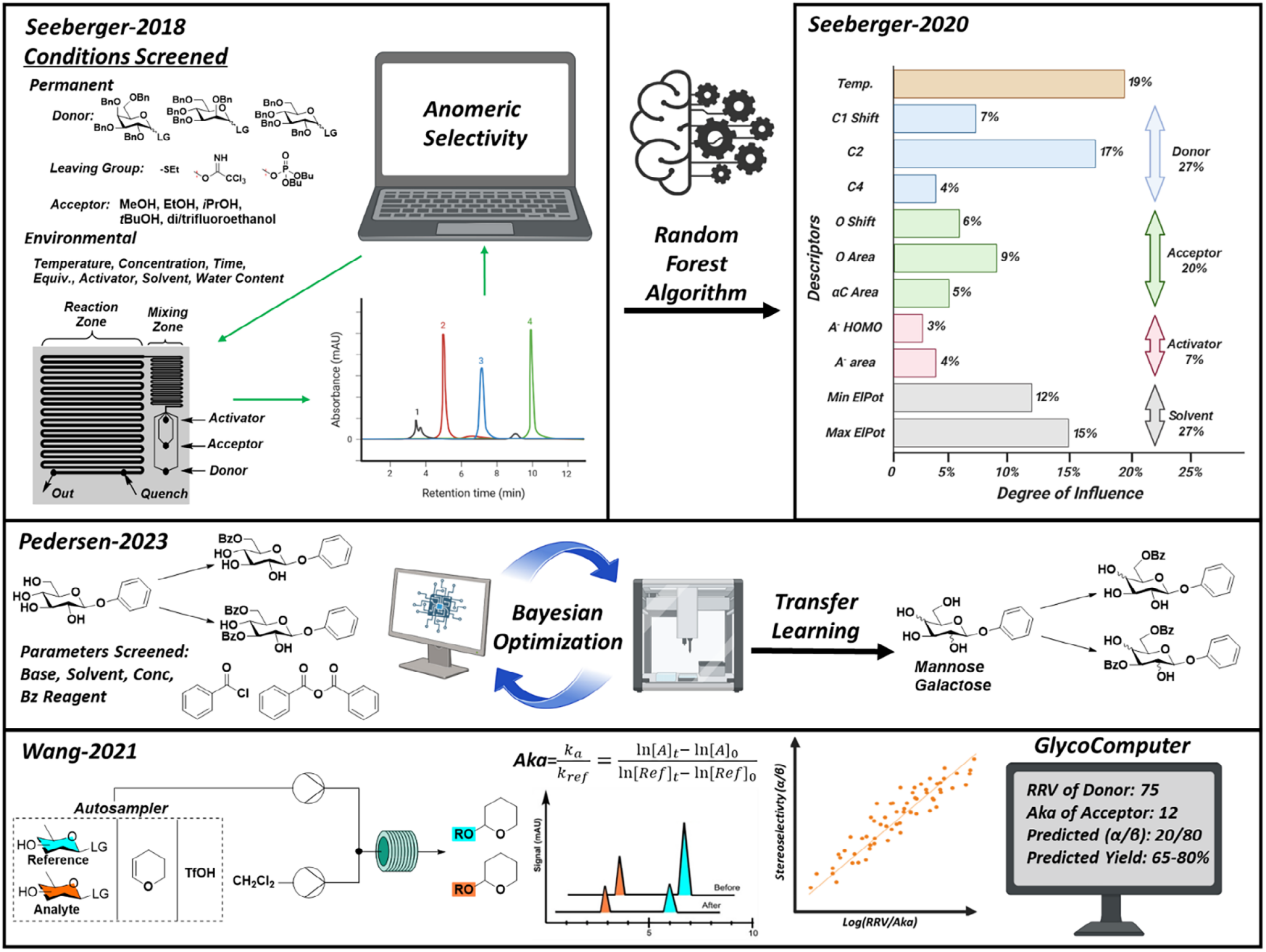
Carbohydrate reaction prediction using automated systems for the collection of homogenous data.

Automated HTE platforms are a means to overcome the scarcity and heterogeneity in glycosylation data (Figure 6). Our laboratory developed an automated microfluidic‐based screening platform to compare the effects of varying both continuous (temperature, acceptor stoichiometry, and presence of water) and discrete (solvent, type, and stereochemistry of leaving group, type of activator and acceptor nucleophilicity) parameters on the stereoselectivity of several glycosylations.^[^ ^75^ ^]^ The data obtained in this way were used to train a random‐forest model on manually selected structural descriptors to derive the most influential factors on stereoselectivity.^[^ ^75^ ^]^ Wang introduced an HPLC‐based automated platform to establish a single nucleophilicity metric for an array of acceptors.^[^ ^102^ ^]^ The GlycoComputer software successfully predicts glycosylation yields and stereoselectivity by statistically analyzing the properties of donors, acceptors, activation systems, and solvents, specifically comparing the RRV of donors and their introduced nucleophilic constant of acceptors.^[^ ^103^ ^]^ Pedersen in a closed‐loop Bayesian optimization improved protecting group manipulations in regioselective benzoylations of unprotected glycosides and a transfer learning method was applied to accelerate the optimization process of regioselectively protecting new substrates.^[^ ^104^ ^]^

## 6 Summary and Outlook

**Figure 7:**
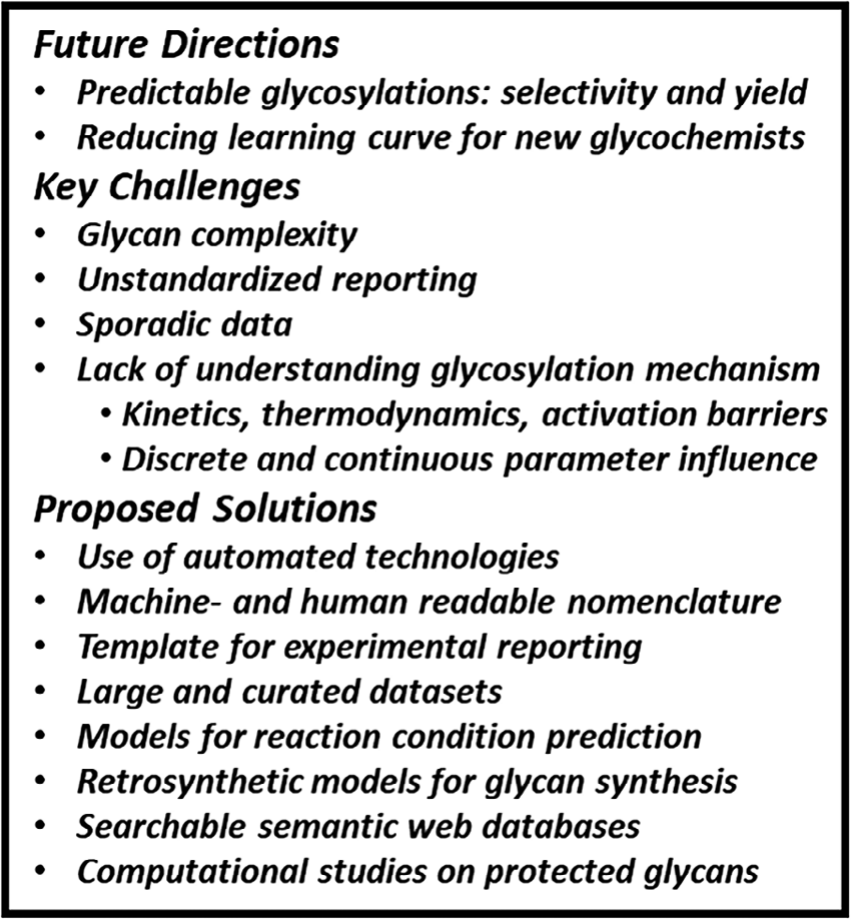
Current challenges and proposed solutions for the digitialization of glychemistry.

Quick access to defined complex glycans is needed to procure substrates that enable molecular glycobiology (Figure 7). Digital tools can only be employed efficiently when the current experimental practices and reporting are revised. Reporting standards need to be decided upon and then implemented, similar to MIRAGE (Minimum Information Required for A Glycomics Experiment) standards set up by the glycomics community.^[^ ^105^ ^]^ As mentioned by Crich and Andreana, big data approaches need to include concentration, and glycosylations should be conducted at a well‐defined temperature.^[^ ^34^ ^]^ Along with structured data generation from new experiments and mining the literature, fundamental work concerning reaction order, rate, and barriers of activation for glycosylations and auxiliary chemical manipulations is pivotal for designing efficient automated assembly protocols and developing accurate retrosynthetic predictor models.

## Conflicts of Interest

The authors declare no conflict of interest.

## Data Availability

Data sharing is not applicable to this article as no new data were created or analyzed in this study.
